# Effect of *Moringa oleifera* Seed Powder on the Clarity, Colloidal Particles, and Nutritional Contents of Cashew Apple Juice

**DOI:** 10.1155/2020/4380407

**Published:** 2020-05-12

**Authors:** Cajethan U. Ugwuoke, Felicitas G. Ugwuoke, Benedicta A. Omeje, Godwin E. Eze, Nnennaya S. Monwuba, Florence O. Ifeanyieze, Juliana A. Ukonze

**Affiliations:** Department of Agricultural Education, University of Nigeria, Nsukka, Nigeria

## Abstract

Cashew apple juice is a good source of vitamins, minerals, and other nutrients. Cashew apples are being wasted due to lack of processing and are not much cherished because of its astringency. Some of the available clarifying agents are costly and are mainly imported. The study was, therefore, aimed at finding out the effect of *M. oleifera* seed powder on the clarity and phytochemical and nutritional contents of cashew apple juice. The study adopted an experimental design where 5 g, 10 g, 15 g, and 20 g of *M. oleifera* seed powder were used to clarify 250 cm^3^ of cashew apple juice. The setups were filtered using a filter paper after standing for an hour to get the clarified juice. The clarity of each filtrate was determined using an atomic absorption spectrometer (AAS). Similarly, the tannin, lignin, pectin, protein, starch, calcium, zinc, copper, magnesium, and vitamin C were determined following the procedure of the Association of Official Analytical Chemists (AOAC). Results showed that cashew apple juice clarified with 10 g/250 cm^3^ gave the best clarity. The colloidal particle contents of cashew apple juice were significantly reduced by the use of *M. oleifera* seed powder. It was equally found out that the clarified cashew apple juice contained an appreciable amount of nutrients. The flavour, colour, and general acceptability of the clarified cashew apple juice were very much liked by the panellist while the aroma was moderately liked. The study recommended that *M. oleifera* seed powder should be used for clarifying cashew apple juice and be included at the rate of 10 g/250 cm^3^.

## 1. Introduction

Cashew apples (*Anacardium occidentale*) are generally grown in the tropics. Cashew is adaptable to a wide range of ecological differences as it grows in warm regions, well adapted to seasonally wet and dry tropical climates, and it gives reasonably good yield on well-drained, light soils [1]. The levels of sugars present in cashew apple juice are sufficient to be used as a substrate for the production of wine, alcohol, and vinegar [2]. The apple juice is a rich source of carbohydrates, minerals, vitamins, amino acids, carotenoids, phenolics, organic acids, and antioxidants [3]. However, farmers do not have the skills to process the apple into different forms; hence, the apples are being wasted [4]. Besides, people find it difficult to eat fruits because of the astringency [2]. Astringency is caused by the interaction of the tannins present in the cashew apple juice and the salivary proteins and glycoproteins leading to drying and puckering sensation in the mouth [5]. Astringency in cashew apple juice can only be removed through clarification. Clarification is a process by which the semistable emulsion of colloidal plant carbohydrates that support the insoluble cloudy material of a freshly pressed juice is broken. Clarification of natural cashew pulp and hydrolysed pulp using the processes of microfiltration and ultrafiltration increases the luminosity of the clarified juice [6, 7]. In the case of enzymatic clarification, enzyme concentration is the most important factor influencing clarification. According to [8], the increase in polygalacturonase concentration increased the clarity of apple juice by exposing part of the positively charged protein beneath, thus reducing electrostatic repulsion between cloud particles which cause these particles to aggregate to larger particles and eventually settle out. For those authors, decolourized and partially purified enzymes improved the clarity of the juice as well as reduced the amount of some of the haze active phenolics, but a high clarity is rare without ultrafiltration and fining agents like gelatin/bentonite [9]. Fruit juices are naturally cloudy, yet in different degrees especially due to the presence of polysaccharides (pectin, cellulose, hemicelluloses, lignin, and starch), proteins, tannins, and metals [9, 10].

Juice clarification is a very important process of the juice production industry as it enhances the acceptability of the product [9]. In the clarification process, a semistable emulsion of colloidal plant carbohydrates that support the insoluble cloud material of a freshly pressed juice is broken such that the viscosity is dropped. For clear juices, complete depectinization (removal of segments) by the addition of enzymes, fine filtration, or high-speed centrifugation will be required to achieve visual clarity [11]. Nowadays, several methods are used for juice clarification and such include enzymatic clarification, ultrafiltration, centrifugation, earth filtration, and cross-flow membrane filtration [12]. Enzymatic treatment for juice extraction is most commonly used now. Their main purposes are to increase the extraction of juice from raw material, increase processing efficiency (pressing, solid settling, or removal), and generate a final product that is clear and visually attractive. Nonenzymatic clarification involves breaking the emulsion by other means, the most common of which is heat. Other techniques include the addition of gelatin, casein, and tannic acid-protein combinations [6]. Consequently, many clarifying agents are used such as Sago (a refined commercial preparation of starch from cassava (*Manihot esculenta*), starch, gelatin, and polyvinylpyrrolidone (PVP) [13], but *Moringa oleifera* seed powder has never been used. Most of the methods used to remove tannins from cashew apple are costly and several clarification agents have to be imported into developing countries [7, 13]. To improve the acceptability of cashew products, it is necessary to find an efficient, locally available, and natural clarifying agent to remove tannins and other colloidal particles to minimize cashew apple wastage. *Moringa oleifera* seed powder is known to be a good coagulant for water purification and has never been used for cashew apple juice clarification.

Optimum performance in the removal of solids and turbidity of groundwater was achieved at a low speed of 96 rpm, and 2 hours using *Moringa oleifera* seed extract [14]. The study of [15] revealed that 100 mg/L of *Moringa oleifera* seed powder gave the best result for wastewater treatment as the treated water showed the lowest value of turbidity, pH of 7, and the lowest value of conductivity. The study is, therefore, aimed at determining the effects of *M. oleifera* seed powder on the clarity, phytochemical content, nutritional composition, and sensory qualities of cashew apple juice.

## 2. Materials and Methods

### 2.1. Experimental Procedure

Mature and dry *Moringa* pods were harvested from the *Moringa* plants present in the vicinity of the researchers. The pods were broken to get the kernels which were further broken to remove the outer shell covering the seeds. The whitish seeds were further dried under the sun. The dried seeds were crushed using a grinding machine to get the powder.

Similarly, cashew apples were purchased from Ogige market and transported to the Department of Nutrition and Dietetics Analytical laboratory, University of Nigeria, Nsukka, located at 6.84°N, 7.37°E. The cashew fruits were cut after removing the nuts and mashed, and the juice was extracted by pressing the mash. The juice was stored in a refrigerator at 40°C for 24 hours. The absorbance was taken to measure the cloudiness or clarity of the raw juice and yielded a value of 43 abs. The colloidal particle contents of the raw cashew juice were tested before and after the clarification.

Then, clarification was done using 5 g, 10 g, 15 g, and 20 g of *M. oleifera* seed powder in 250 cm^3^ of cashew apple juice replicated three times. The setups were filtered using filter paper after standing for an hour to get clarified juice. A known quantity of each filtrate was placed in an atomic absorption spectrometer (AAS) to get the absorbance readings. The readings were taken three times. The sample with the lowest reading gave the best clarity and was used to determine the phytochemical and nutritional contents of the clarified juice following the procedure of [16]. The laboratory analysis was carried out in the Analytical Lab of the Department of Nutrition and Dietetics, University of Nigeria, Nsukka, located at 6.84°N, 7.37°E. A panel of 30 members was constituted and was served with the clarified juice to determine the sensory qualities. Besides, a 9-point hedonic scale and clean drinking water to rinse their mouth each time the juice was taken were served. The hedonic scale had a value ranging from 1 to 9 where 1 represented “dislike extremely” while 9 represented “like extremely.” The copies of the hedonic scale were filled by the panel members and collected by the researchers.

### 2.2. Determination of Lignin

Lignin was determined following the procedure described in [16]. 200 ml of 2NH_2_SO_4_ was added to a 100 ml portion of the cashew juice. This was boiled on a hot plate for 25 min. An empty filter paper was dried in a hot air oven and weighed. The oven-dried filter paper was used to filter the mixture. The purple parches on the filter paper indicated the presence of lignin. The filter paper was dried again in an electric oven and the second reading was taken. The difference between the first and the second readings indicated the amount of lignin present in the sample. The experiment was repeated after clarification and both readings were compared to determine the decrease in the amount of lignin in the clarified juice.

### 2.3. Determination of Pectin

Following the procedure of [17], 200 ml of cashew juice was collected in a 1000 ml beaker and the pectic substances were extracted with 400 ml 0.05 M HCl for 2 hrs at 80–90°C. The mixture was then cooled, the volume was made up to 500 ml, and it was filtered with Whatman No. 4 filter paper. Filtrate about 100 ml was neutralized and then 10 ml 1 M NaOH was added with constant stirring. It was allowed to stand overnight. 50 ml acetic acid (1 M) was added, allowed to stand for 5 min. This was followed by the addition of 25 ml 0.5 M CaCl_2_. It was allowed to stand for 60 min, boiled for 1–2 min, and then filtered through previously prepared filter paper (filter paper wet in hot water dried in an oven at 102°C for 2 hrs, cooled in a desiccator, and weighed in a covered dish). The precipitates were washed with hot water until being free from chloride (tested using 1% silver nitrate). Filter paper containing calcium pectate was dried overnight at 100°C, cooled in a desiccator, and weighed, after which, the percentage of pectin was calculated.

### 2.4. Determination of Tannin

The tannin content of the cashew apple juice was determined following the procedure described in AOAC (2010). 1 ml of the cashew apple juice was mixed with folin-ciocalteu reagent (0.5 cm^3^), followed by the addition of saturated Na_2_CO_3_ solution (1 cm^3^) and distilled water (8 cm^3^). The reaction mixture was allowed to stand for 30 min at room temperature. The supernatant was recorded by centrifugation and absorbance was recorded at 725 nm using a UV-visible spectrophotometer. This experiment was repeated after clarification.

### 2.5. Determination of Protein

The micro-Kjeldahl method described by [16] was used in the determination of crude protein, which involved the following stages.

#### 2.5.1. Digestion

One gram of cashew apple juice was digested with a mixture of 20 ml concentrated sulphuric acid, a pinch of copper sulphate, 2 g sodium sulphate, and a pinch of selenium catalyst. The mixture was heated till the black liquid cleared. Heating continued until the sample became completely digested. The digested sample was transferred into a 100 ml volumetric flask, which was weighed in preparation for distillation.

#### 2.5.2. Distillation

Kjeldahl distillation apparatus was used. 10 ml of 4% boric acid mixed indicator was introduced into a conical flask of 100 ml. The conical flask was positioned at the receiving end of the distillation unit using a clamp. 10 ml of the digested cashew apple juice was first introduced into the distillation unit followed by the addition of 10 ml of 50% NaOH gradually. The distillation process lasted for 5 to 10 min, during which ammonia (NH_3_) was trapped in excess boric acid. The presence of ammonia changed the purple colour of boric acid to green which is a common characteristic of an alkaline gas. Ammonia trapped in the boric acid indicator was titrated using 0.01 NHCl. Crude protein content was calculated by the following formula:

(1) $$\begin{array}{c} \%\text{ nitrogen}=\frac{\text{titre }\times \;0.01\;\times \text{ DF }\times \text{ MWN }\times \;100}{\text{weight of the sample }\left(\text{mg}\right)}, \end{array}$$

where DF is the dilution factor (100 ml/5 ml) which is equal to 20. MWN is the molecular weight of nitrogen which is equal to 10.01. Then, *N* × 6.25 was used to convert nitrogen content into protein content.

### 2.6. Determination of Starch

The method of Pearson (1976) as adopted in [16] was used in this experiment. 5 g of cashew apple juice was defatted in a flask with 100 ml of 12% alcohol by shaking, filtering, and washing with 20 ml of industrial alcohol. The defatted residue was washed into a flask with 50 ml of ammonia-free water, heated in boiling water for 20 min, cooled, added 0.1 g of diastase (+water), and maintained at 50–55°C for 2 hours. After it was cooled, it was filtered and the filtrate was made up to 250 ml. 200 ml of the filtrate was mixed with 20 ml HCl (sp. Gravity 1.125) and heated in boiling water for 3 hours. It was cooled and then neutralized with NaOH solution and then made up to 250 ml. The estimation of the reducing sugars using Fehling's solution was carried out and the value obtained was calculated as dextrose (dextrose × 0.90 = starch). This experiment was repeated after clarification.

### 2.7. Determination of the Nutritional Composition of Cashew Apple Juice

Cashew apple juice clarified with 10 g/250 cm^3^ of *M. oleifera* seed powder was used to determine the nutritional composition as it produced the best clarity.

#### 2.7.1. Determination of Calcium

This was determined following the procedure of [16]. 10 g of the ash solution of cashew apple juice was taken into a 250 ml beaker. 1 ml 30% citric acid solution was added followed by 5 ml 5% ammonium chloride solution. This was made up to 100 ml with distilled water and brought to boiling. 10 drops of 0.04% bromocresol green solution were added. This was followed by the addition of a 30% warm saturated 4.2% ammonium oxalate solution. The precipitate formed was dissolved in few drops of concentrated HCl. This was neutralized slowly by the dropwise addition of ammonia (0.88). The beaker was placed on a steam bath for 30 min. The beaker was removed and its content was filtered. The beaker was thoroughly washed and the precipitate was dissolved by passing through 50 ml 2NH_2_SO_4_. This was filtered. The filter paper was rinsed and the filtrate was made up to 100 ml with distilled water. The filtrate was heated to 70–80°C and then titrated with 0.02 N KMnO_4_ solution to a pink that persisted for about 30 seconds:

(2) $$\begin{array}{c} \text{calculation : }1\text{ ml }{\text{KMnO}}_{4}=2\text{.}004\text{ mg}\cdot \text{Ca}. \end{array}$$

#### 2.7.2. Determination of Zinc

Following the procedure of [16], the dithizone method was used to determine the zinc content of cashew apple juice. 10 ml ash solution of cashew apple juice was pipette into a 30 ml test tube. 5 ml acetate buffer was added followed by 1 ml 2% sodium thiosulphate solution. Then, there was an addition of a 5 ml 0.05% dithizone solution. The mixture was left to react for about 5 min after which a 3 ml carbon tetrachloride solution was added. The mixture was separated into two layers of which the upper layer was taken for specification reading at 540 nm:

(3) $$\begin{array}{c} \text{calculation : Zn }\left(\frac{\text{mg}}{100\text{ g}}\right)=\frac{\text{abs of sample }\times \text{ conc}.\text{ standard}}{\text{abs of standard }\times \text{ weight of the sample}}. \end{array}$$

#### 2.7.3. Determination of Copper

The optimized resorcinol method of copper determination described in [18] was adopted in this experiment. 0.6 ml of 0.2 M ammonia was added to a 10 ml ash solution of cashew apple juice. Then, 0.2 ml of 0.1% resorcinol reagent was added, well mixed, and allowed standing at room temperature. The colour was monitored at 450 nm (UV-visible spectrophotometer SL–150) following one hour standing at room temperature. The reaction mixture was incubated in a boiling water bath for 3 min and the reading of the sample was done within 5 min following the cooling to room temperature.

#### 2.7.4. Determination of Magnesium

Following the procedure of [16], 10 ml of ash solution of cashew apple juice was pipetted into a 250 ml beaker. 25 ml pH 10 buffer was added. This was followed by the addition of 25 ml of distilled water. 0.1 g EBT indicator was added and the solution swirled to create a wine-coloured solution that was titrated against 0.01 N EDTA to a clear blue endpoint. The percentage of magnesium was then calculated.

#### 2.7.5. Determination of Vitamin C

The method of [16] was used. 5 g of cashew apple juice was weighed into a flat-bottomed flask and 60 ml TCA/acetic acid solution was added. The mixture was left for about 60 min before it was filtered. The filtrate was made up to 200 ml. 10 ml was taken for titration with 0.05% 2,6-dichlorophenol indophenol. The vitamin C content was calculated as follows:

(4) $$\begin{array}{c} \text{K}=\frac{y\;\times z\;\times \text{DF}}{\text{wt}.\text{ of sample}}, \end{array}$$

where *y* was the titre of the sample. *z* was the figure gotten when 50 mg of the standard vitamin C was divided by its titre value. DF was the dilution factor, and K was then multiplied by a factor of 20 to give vitamin C content in mg/100 g.

### 2.8. Methods of Data Analysis

The data collected were analysed using mean and standard deviation to answer the research questions. Similarly, *t*-test statistic and analysis of variance (ANOVA) were used to test the null hypotheses at 0.05 level of significance. A null hypothesis was accepted when the probability value was equal to or greater than 0.05 and was rejected when the probability value was less than 0.05. The data collected from the responses of the panel members were analysed using mean. The decision on the mean was taken using a real limit of number based on the grand mean. Any sensory attribute with a mean value ranging from 9.50 to above was interpreted as “like extremely,” 8.50–9.49 was interpreted as “like very much,” 7.50–8.49 as “like moderately,” 6.50–7.49 as “like slightly,” among others.

## 3. Results and Discussion

### 3.1. Clarity of Clarified Cashew Apple Juice

Cashew apple juice clarified with 10 g *M. oleifera* seed powder had the best clarity with the absorbance value of 0.321 (Table 1). The highest turbidity was recorded in cashew apple juice clarified with 5 g *M. oleifera* seed powder with the absorbance value of 0.405. However, after attaining the best clarity with 10 g *M. oleifera* seed powder, turbidity increased with an increase in the inclusion level of *M. oleifera* seed powder (Table 1). The turbidity of the clarified cashew apple juice was statistically higher in that it clarified with 5 g *M. oleifera* seed powder than others. The best clarity recorded in cashew apple juice clarified with 10 g *M. oleifera* seed powder was not significantly different (*p* > 0.05) from the clarity of those clarified with 15 g and 20 g *M. oleifera* seed powder (Table 1).

The result was comparable to [19] who discovered that 12.0 g/1000 ml dose of *M. oleifera* seed powder as coagulant gave the highest water clarity by reducing the turbidity to log_10_^1.32NTU^. Similarly, the result was in line with the study of [20] who found out that *M. oleifera* seed powder significantly improved the clarity of water by reducing the turbidity. The study [21] reported that *M. oleifera* seed powder improved the quality of drinking water by reducing the turbidity from 339NTU to 4.10NTU. The mechanism of coagulation of colloidal particles with the powdered seeds of *M. oleifera* according to [22] consisted of adsorption and neutralization of the colloidal positive charges that attract the negatively charged impurities. This mechanism was different from other clarifying agents in removing the colloidal particles that increased the turbidity of cashew apple juice. While PVP chelates the colloidal particles and sediment them at the bottom, starch removes the particles through the technique of [23].

### 3.2. Colloidal Particles of Clarified Cashew Apple Juice

The tannin content of raw cashew apple juice was found to be 0.02% and drastically reduced to 0.004% with the elimination of 80% after clarification with 10 g *Moringa oleifera* seed powder (Table 2). The amount of lignin present in raw cashew juice was found to be 0.35% but declined to 0.05% with the elimination of 86% after clarification with 10 g of *M. oleifera* seed powder. In the same vein, it was found that 0.95% of pectin was present in raw cashew juice but was reduced to 0.30% with the elimination of 68% after clarification with 10 g *M. oleifera* seed powder. The result also revealed that 0.55% of starch was contained in raw cashew juice but was reduced to 0.15% with the elimination of 73% after clarification with 10 g *M. oleifera* seed powder. The protein content of raw cashew juice was found to be 0.74% but was reduced to 0.00% after clarification with 10 g *M. oleifera* seed powder inclusion (Table 2). The result equally showed that there was a statistically significant decrease (*p* < 0.05) in the colloidal particle contents of cashew apple juice clarified by the inclusion of *M. oleifera* seed powder (Table 3). Tannin was the major cause of astringency in raw cashew apple juice which was significantly reduced by clarifying the juice with *M. oleifera* seed powder. Similarly, lignin, pectin, and starch were the main cause of cloudiness in cashew apple juice. The result of this study appeared to be better than using cassava and rice starch for clarification as reported in [7] who discovered that cassava starch at 6.2 ml/l decreased tannin content at 34% while rice starch at 10 ml/l decreased tannin content at 42.14%. The mechanism of colloidal particle reduction in cashew apple juice using *M. oleifera* seed powder according to [19] was due to its positively charged proteins which acted like magnets attracting predominantly negatively charged particles. The authors emphasized that under proper agitation, these bound particles grew in size to form the flocculates which settled by gravity and could easily be removed by filtration. This accounted for the entire removal of protein from the clarified cashew apple juice.

### 3.3. Nutritional Composition of the Clarified Cashew Apple Juice

Cashew apple juice was found to contain 155 mg/100 g vitamin C, 0.291 mg/100 g copper, 57.6 mg/100 g calcium, 0.21 mg/100 g zinc, and 16.32 mg/100 g magnesium after clarification with *M. oleifera* seed powder (Table 4). There was no statistically significant difference (*p* > 0.05) in the nutritional composition of cashew apple juice produced using *M. oleifera* seed powder for clarification (Table 5). The result was comparatively better than [24] who clarified cashew apple juice with rice gruel and found to contain vitamin C (109.41 mg/100), calcium (10.1 mg/100 g), and magnesium (6.1 mg/100 g). Unclarified cashew apple juice was found to contain 152.9–215.1 mg/l of magnesium, 5.1 mg/l of copper [25], and 370.9–480.3 mg/100 g of vitamin C [2]. In support of the findings, [26] found out that unclarified cashew apple juice contained 231.4 mg/100 ml of vitamin C, 43.0 mg/100 ml of calcium, 10.92 mg/100 ml of magnesium, and 0.08–0.08 mg/100 ml of copper and zinc. There was a substantial decrease in the nutrient of cashew apple juice after clarification.

### 3.4. Sensory Qualities of Clarified Cashew Apple Juice

Findings indicated that the panellists like very much the flavour (8.20, 1.76), colour (8.35, 1.70), general acceptability (8.42, 1.34), and moderately like the aroma (7.40, 1.25) of clarified cashew apple juice (Table 6). The acceptability of the flavour could be attributed to the reduction in the astringency caused by the tannin content of the raw cashew apple juice. This was in line with the study of [27] who found out that cashew apple juice was not consumed like other fruit juices due to its characteristic astringent taste, which caused a biting sensation of the tongue and throat.

## 4. Conclusions

*M. oleifera* seed powder increased the clarity of cashew apple juice with the optimum result being achieved at 10 g/250 cm^3^. *M. oleifera* seed powder significantly decreased the tannins, lignin, pectin, and starch that contributed to the astringency and cloudiness of the raw juice. After clarification with *M. oleifera* seed powder, the clarified juice still retained a significant amount of vitamin C, copper, calcium, zinc, and magnesium. Consumers liked very much the flavour, colour, and general acceptability and moderately liked the aroma of the clarified juice.

## 5. Recommendations

Based on the findings, the following recommendations were made:

1. *M. oleifera* seed powder should be used for cashew apple juice clarification as it is locally available and to reduce the use of expensive imported clarifying agents
2. *M. oleifera* seed powder should be used at the rate of 10 g/250 cm^3^ of cashew apple juice to maximize clarity and reduce astringency

## Figures and Tables

**Table 1:** Mean and ANOVA analysis of the effects of *M. oleifera* seed powder on the clarity of cashew apple juice.

Inclusion levels	1^st^ reading (absorbance)	2^nd^ reading (absorbance)	3^rd^ reading (absorbance)	Average (absorbance)	Std. deviation	Std. error
5 g *M. oleifera*	0.323	0.41	0.48	0.405^b^	0.079	0.045
10 g *M. oleifera*	0.363	0.28	0.318	0.321^a^	0.040	0.023
15 g *M. oleifera*	0.365	0.333	0.351	0.350^a^	0.016	0.009
20 g *M. oleifera*	0.383	0.36	0.404	0.382^a^	0.022	0.013

**Table 2:** Mean percentage analysis of the effects of *Moringa oleifera* seeds powder on the colloidal particles of cashew juice.

Phytochemicals	Before (%)	After (%)	Difference (%)	% Removed
Tannin	0.02	0.004	0.016	80
Lignin	0.35	0.05	0.3	86
Pectin	0.95	0.3	0.65	68
Starch	0.55	0.15	0.4	73
Protein	0.74	0	0.74	100
**Total**	**2.61**	**0.504**	**2.106**	

**Table 3:** *t*-test analysis of the effects of *M. oleifera* seed powder on the colloidal particle contents of cashew apple juice.

One-sample test						
*t*		df	Sig. (2-tailed)	Mean difference	95% confidence interval of the difference	
					Lower	Upper
Before	3.259	4	0.031	0.52200	0.0773	0.9667
After	1.779	4	0.150	0.10080	−0.0565	0.2581

**Table 4:** Mean analysis of the nutritional compositions of cashew apple juice clarified using *M. oleifera* seed powder.

	Trial 1 (mg/100 g)	Trial 2 (mg/100 g)	Trial 3 (mg/100 g)	Mean (mg/100 g)
Vitamin C	158.9	153.2	155.3	155.8
Copper	0.236	0.285	0.352	0.291
Calcium	53.5	60.8	58.5	57.6
Zinc	0.19	0.23	0.21	0.21
Magnesium	15.87	17.31	15.78	16.32

**Table 5:** ANOVA analysis of the nutritional compositions of cashew apple juice clarified using *M. oleifera* seed powder.

ANOVA					
Nutrients	Sum of squares	df	Mean square	*F*	Sig.
Between groups	0.981	2	0.49	<0.01	1.00
Within groups	51817.569	12	4318.131		
Total	51818.550	14			

**Table 6:** Mean analysis of the sensory qualities of cashew apple juice clarified using *M. oleifera* seed powder.

Attributes	Mean	Std. deviation	Decision
Flavour (taste)	8.20	1.76	Like very much
Colour	8.35	1.70	Like very much
Aroma (smell)	7.40	1.25	Like moderately
General acceptability	8.42	1.34	Like very much

## Data Availability

The data used to support the findings of this study are available from the corresponding author upon request.
